# Species Distribution and Determinants of *Candida* Urinary Tract Infections: A 10-Year Retrospective Study in a Tertiary Hospital

**DOI:** 10.3390/medicina62050921

**Published:** 2026-05-09

**Authors:** Nada S. Alghamdi, Sakinah H. Alessa, Fatemah A. Almousa, Zainab A. Alkhamis, Shaima A. Alkhardawi, Hawraa A. Alsalem, Nehal Hosin, Maher S. AlQurashi, Ayman A. El-Badry

**Affiliations:** 1 Department of Microbiology, College of Medicine, Imam Abdulrahman Bin Faisal University, Dammam 31441, Saudi Arabia; nmhosin@iau.edu.sa (N.H.); msalqurashi@iau.edu.sa (M.S.A.); aaelbadry@iau.edu.sa; 2 College of Medicine, Imam Abdulrahman Bin Faisal University, Dammam 31441, Saudi Arabia; s.cina.s@hotmail.com (S.H.A.); fatemahalmousa17@outlook.com (F.A.A.); zainabazizkh9@gmail.com (Z.A.A.); shaima.alkhardawi@gmail.com (S.A.A.); hawra1602@gmail.com (H.A.A.)

**Keywords:** candiduria, funguria, *Candida* epidemiology, urinary tract infection

## Abstract

*Background and Objectives*: Candiduria is a common health problem especially among hospitalized patients. In the era of rising azole resistance, evidence from Saudi Arabia remains limited concerning *Candida* species. This study aimed to assess the prevalence and risk factors of *Candida* isolated from urine culture and to explore species distribution in relation to clinical characteristics. *Materials and Methods*: We retrospectively reviewed 188353 urine samples from 2013 to 2023. Using medical records, data on age, gender, hospitalization status, and urine sample type were collected. Identification of *Candida* species was performed by VITEK Mass Spectrometry (bioMerieux Inc.). Binary logistic regression analysis was used to identify predictors of candiduria. A *p* value below 0.05 at a 95% CI was considered statistically significant. *Results*: A total of 1667 urine samples with significant *Candida* growth were reported. It accounted for 0.88% of all organisms grown from urine culture and 30% of *Candida* grown from various body sites. *Candida albicans* was the most frequently identified species (n = 920, 55.2%), followed by *C. tropicalis* (n = 374, 22.4%), *C. krusei* (n = 80, 4.8%), *C. glabrata* (n = 78, 4.7%), and *C. parapsilosis* (n = 41, 2.5%). However, the rate was not stable throughout the years, and non-albicans *Candida* (NAC) was often the most prevalent. Female gender was the strongest predictor of candiduria (OR and AOR 1.81, 95% CI 1.46–2.25), whereas significantly lower odds were seen in elderly patients and in random urine specimens. The species distribution of NAC did not seem to change with age, gender, type of specimen, or hospitalization status. *Conclusions*: Among all *Candida* spp. isolated in the lab, 30 out of every 100 originated from urine culture, with a significant risk associated with females. The increasing prevalence of emerging *Candida* species in tertiary care settings can provide clinicians with valuable insights for the diagnosis and management of *Candida* UTI.

## 1. Introduction

Candiduria, defined as significant growth of *Candida* species ≥ 10^5^ CFU/mL of urine, is a growing global health problem. Urinary tract infections (UTIs) caused by *Candida* species are opportunistic in nature, primarily affecting immunocompromised individuals and hospitalized patients. Many risk factors play a role in candiduria such as major abdominal procedures, prolonged hospitalization, extremes of age, female gender, extensive use of wide-spectrum antibiotics, and immunosuppressive therapy [1,2]. *Candida* in urine is of clinical significance in critically ill individuals where it was linked to higher mortality compared to those without candiduria [3]. They are second only to *Escherichia coli* in combined medical–surgical ICUs [4]. In the hospital setting, it represents 10–15% of nosocomial urinary tract infections and patients are often asymptomatic, making prompt diagnosis very challenging [5]. Community-acquired candiduria is mostly observed in bedridden patients, those with diabetes mellitus, or those undergoing antimicrobial therapy and usually present with symptoms similar to that of bacterial UTIs [6].

Among *Candida* species, *Candida albicans* is the major colonizer of the urinary tract and the most common etiological agent of candiduria. Their capacity to adhere to catheters, forming biofilms, and to invade host tissues by means of production of extracellular enzymes has shifted their role from commensals to pathogens [1]. These virulence factors have also been reported in NAC species. In recent decades, a worldwide shift towards non-albicans *Candida* (NAC) has been noticed [7]; many were reported as a causative agent of nosocomial UTIs [1]. The poor outcomes associated with the increasing incidence of NAC spp. are linked to their inherent resistance to fluconazole and emerging resistance to other antifungal classes [8,9,10,11].

Therefore, *Candida* speciation in patients with UTIs is necessary for the initiation of appropriate therapy. In this work, we retain the traditional *Candida* nomenclature for consistency with the existing literature. Recent taxonomic updates reclassify *Candida glabrata* as *Nakaseomyces glabratus*, *Candida krusei* as *Pichia kudriavzevii*, *Candida guilliermondii* as *Meyerozyma guilliermondii*, and *Candida lusitaniae* as *Clavispora lusitaniae*.

Also, isolation of *Candida* species in the laboratory can sometimes be difficult to interpret. It may represent mere contamination during collection or life-threatening disseminated candidiasis [12]. Under certain conditions, the clinical picture can shift from one manifestation to another. For instance, *Candida* colonization may progress to *Candida* infection if the host factors are changed. Observing trends of *Candida* species is essential for understanding the associated infection and predicting the severity. Due to the current ongoing conflicts in clinical interpretation of candiduria, along with the emergence of NAC species, it is important to conduct studies exploring its worldwide epidemiological pattern. Laboratory data collected from such studies may inform more targeted therapeutic approaches specific to each region. Our study presents 10-year data on *Candida* species isolated from urine cultures; the aims were (a) to assess the prevalence and risk factors of candiduria, and (b) to explore the frequency of species distribution in relation to age, gender, type of urine specimen, and hospitalization status.

## 2. Materials and Methods

### 2.1. Study Design and Patient Population

A retrospective analysis was performed on all *Candida* species isolated from urine cultures submitted to the microbiology laboratory of a 550-bed tertiary hospital in Saudi Arabia. It was carried out on data collected during the period between April 2013 and April 2023. After removal of the duplicates, data on patients’ gender, age, hospital ward/outpatient department, type of specimen, and *Candida* species were extracted from the patients’ medical records. Inclusion criteria comprised all urine samples showing significant *Candida* growth (≥100,000 CFU/mL) during the study period, while samples with no growth or with *Candida* counts below 100,000 CFU/mL were excluded. The isolates were considered different when the samples for the same patient yielded a different *Candida species* or occurred more than 30 days apart.

### 2.2. Identification of the Isolates

To minimize the risk of contamination, local standardized collection protocols were strictly followed for all patients. Urine specimens from non-catheterized patients were collected in sterile, properly labeled screw-capped containers with a minimum volume of 5 mL. Urine from catheterized patients was aseptically obtained from the distal end of a rubber catheter using a sterile 21–23-gauge needle and a 5 mL syringe.

The standard microbiology laboratory protocol was followed during the processing of urine samples. Collected specimens were inoculated within 2 h of collection on MacConkey, Columbia Nalidixic Acid (CNA), and cysteine–lactose–electrolyte-deficient (CLED). Sabouraud dextrose agar is not part of the standard urine culture workflow. Each specimen was taken using a 0.001 calibrated loop and inoculated on each plate, then incubated aerobically at 37 °C for 24 h. The number of colonies grown on CLED media was multiplied by 1000 to determine CFUs. Growth of *Candida* on urine culture was considered significant when the yeast counts were ≥10^5^ CFU/mL. To ensure accurate identification, purity plates were prepared on sheep blood agar from *Candida* recovered on CNA, and a single pure colony was subsequently identified to the species level by using VITEK Mass Spectrometry microbial identification system (BioMérieux, Marcy-l’Étoile, France) as per the manufacturer’s protocol.

### 2.3. Statistical Analysis

Statistical analysis was performed using SPSS software Version 29.0.2.0 (20). Numerical data were checked for normality with the Shapiro–Wilk test and showed a non-normal distribution; therefore, median and range were used. Categorical data were analyzed by using the chi-square test. *p* values below 0.05 were considered statistically significant. In addition, univariate and multivariate analyses were performed to determine the significant predictors of *Candida albicans*, with corresponding crude and adjusted odds ratios as well as 95% confidence intervals to measure the strength of associations.

## 3. Results

### 3.1. Demographic Data

In the 10-year study period, a total of 1667 urine samples with positive *Candida* growth were recovered. Table 1 shows patient and sample characteristics. In this analysis, the male-to-female ratio was 1:25. The median age of the patients was 53 years. Almost 49% (n = 811) of *Candida* growth in culture was isolated from adults, followed by the elderly group (n = 745, 45%). Candiduria was less frequently seen in pediatrics under the age of 18 years. Most samples were obtained from hospitalized patients; 35.5% of those were in medical wards followed by 26.6% from critical patients in intensive care units. *Candida* species were predominantly isolated from catheterized samples (n = 674, 40%) and were less seen in sterile bladder specimens collected by suprapubic aspiration.

### 3.2. Prevelance and Temporal Trend of Candiduria

During the 10-year study period, a total of 188,353 urine samples were sent to the microbiology laboratory. Candiduria was detected in 1667 of those samples. Prevalence was calculated as 0.88% of all organisms grown from urine samples submitted during the study period, and 30% of all *Candida* species isolated from all body sites (n = 5459).

A total of nine different types of *Candida* species isolated from growth on urine culture were reported; others were released as *Candida* species. These include *Candida albicans* (n = 920, 55.2%), *C. tropicalis* (n = 374, 22.4%), *C. krusei* (n = 80, 4.8%), *C. glabrata* (n = 78, 4.7%), *C. parapsilosis* (n = 41, 2.5%), *C. lusitaniae* (n = 21, 1.26%), *C. utilis* (n = 10, 0.60%), *C. lipolytica* (n = 2, 0.12%), and *C. guilliermondii* (n = 1, 0.06%). None of the NAC isolated in 2013 were identified at the species level and were reported as *Candida* species in the record. These isolates accounted for 8.4% of all isolated *Candida*.

**Figure 1:**
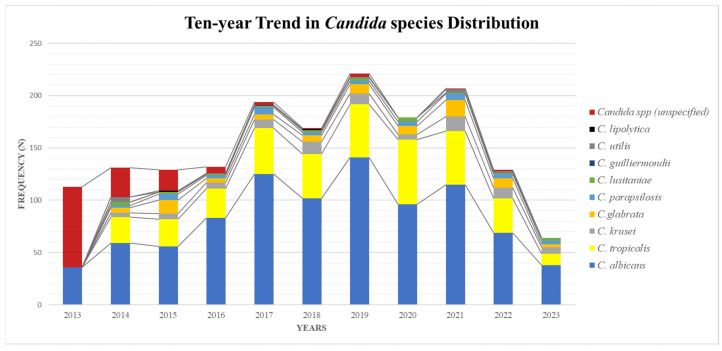
Distribution of *Candida* species isolated from urine specimens from 2013 to 2023.

Across the study period, the prevalence of candiduria showed little variation, maintaining a stable pattern till the end of 2016, where a marked increase in the number of cases was noticed (Figure 1). The increase was observed for both *Candida albicans* and emerging NAC simultaneously. A high number of cases were reported in the year 2019 (n = 221), then in 2021 (n = 207) and 2017 (n = 194). Throughout the study period, *Candida albicans* was the most prevalent species except in 2013 and 2015 where non-albicans *Candida* predominated. Within NAC, *C. tropicalis* was more frequently observed in 2020 (35%) then in 2015 (20%), and *C. glabrata* was the major *Candida* group in 2015 (10%) (Figure 1).

### 3.3. Factors Associated with the Occurrence of Candiduria

The regression analysis in Table 2 demonstrates several key predictors of *Candida albicans* positivity; in univariate analysis, samples obtained from patients older than 60 years had significantly lower odds of infection compared with those from patients aged 18 years or younger (OR 0.57, 95% CI 0.46–0.69, *p* < 0.001), a relationship that remained stable after adjustment for nationality and location (AOR 0.56, 95% CI 0.46–0.69, *p* < 0.001), indicating an independent age effect. Gender was the strongest predictor, with females exhibiting nearly double the odds of *C. albicans* positivity compared with males in both univariate and adjusted models (OR and AOR 1.81, 95% CI 1.46–2.25, *p* < 0.001), highlighting a robust and biologically plausible association. Nationality showed no meaningful influence, as samples from non-Saudi patients had similar odds to those from Saudi patients in both analyses (OR 0.96, 95% CI 0.76–1.23, *p* = 0.760; AOR 0.96, 95% CI 0.76–1.23, *p* = 0.761), and patient location (inpatient vs. outpatient) likewise demonstrated no significant effect (OR and AOR 1.09, 95% CI 0.89–1.35, *p* = 0.375).

There was considerable variation across specimen types. Random urine samples had a lower odd of *C. albicans* in both univariate (OR 0.67, 95% CI 0.51–0.88, *p* = 0.003) and adjusted models (AOR 0.67, 95% CI 0.51–0.88, *p* = 0.003). Midstream urine samples showed a slight increase in odds that nearly reached statistical significance after adjustment (AOR 1.26, 95% CI 0.98–1.63, *p* = 0.071), while catheter samples did not show a significant association (AOR 1.13, 95% CI 0.72–1.79, *p* = 0.589). The adjusted model found that female gender and specimen type were the most consistent independent predictors of *C. albicans* positivity. The lower odds observed in older adults persisted after adjustment for confounders, suggesting a complex epidemiological pattern that warrants further study.

### 3.4. Association Between Candida Species in Patients with UTI and Clinical Characteristics

The distribution of *Candida* species was further analyzed according to the patients’ characteristics including age group, gender, location at the time of specimen collection, and the type of urine specimen submitted to the microbiology laboratory (Table 3, Figure S1). The frequency of *Candida* isolates reported in samples from females was higher than those from males (n = 1190, 71.4% vs. n = 477, 28.6%) among all *Candida* species over the years with statistical significance (*p* value < 0.05). More NAC species were seen in samples from female patients compared with those from males.

The highest frequency of *Candida* isolation from urine was in samples from the adult age group [18–60 years] (n = 811, 48.7%), followed by those from older patients (n = 745, 44.7%), with statistical significance (*p* value < 0.05) (Figure S1B). In both age groups, *Candida albicans* was the predominant species, followed by *Candida tropicalis* and other NAC species. In neonates, only 13 urine samples were reported, with 10 isolates identified as *C. albicans*, 2 as *C. tropicalis*, and 1 as *C. guilliermondii*. Similarly, 27 urine samples were reported in infants, including 18 *C. albicans* isolates, 6 *C. tropicalis*, and 3 other NAC species. The distribution of *Candida* in samples from patients under 18 years old remained relatively consistent (Figure S1B).

*Candida* cases were more common in samples from hospitalized patients (n = 1141, 68%) compared to those from outpatient departments (n = 526, 31%) (*p* value < 0.05) (Figure S1C). The highest number of *Candida* cases was recorded in 2021 for samples from inpatients and in 2019 for samples from outpatients. In both groups, *Candida albicans* (n = 920) followed by *Candida tropicalis* (n = 373) were the most frequently isolated species, surpassing all other non-albicans *Candida* (NAC) species throughout the years, except in 2013. The patterns of NAC species differed between samples from hospitalized and non-hospitalized patients, with *Candida glabrata* being more commonly reported in inpatients (n = 49) across all years except 2015 (Figure S1C).

The distribution of *Candida species* across different types of urine specimens was analyzed. The highest number of Candida isolates came from samples obtained from catheterized patients (n = 674, 40.4%) with statistical significance (*p* value < 0.05). (Figure S1D). Urine specimens obtained directly from the bladder via a suprapubic catheter had the fewest Candida isolates during the study period (n = 90). Consistent with other parameters, *Candida albicans* was the most frequently isolated species in all years (n = 920), except in 2013. Among NAC species, *Candida tropicalis* was the most common (n = 373), followed by *Candida krusei* (n = 80) and *Candida glabrata* (n = 78) (Figure S1D).

## 4. Discussion

Candiduria is becoming an increasingly significant health issue, contributing to most fungal urinary tract infections. Our institution has previously studied the clinical and microbiological characteristics, including antifungal susceptibility testing of *Candida* growth from blood culture [13]. However, data on prevalence, risk factors, and species distribution of *Candida* isolates from urine sample are lacking. Therefore, the decision to conduct local surveillance to monitor these important microorganisms was taken. In this cross-sectional retrospective analysis, data were collected from 2013 to early 2023, which explains the lower number of *Candida* isolates in 2023 compared to previous years.

During the 10-year study period, *Candida* isolates from urine specimens represented almost 1% of all organisms grown from urine culture and 30% of all *Candida* isolated from any site (including blood, urine, respiratory specimens, skin/wound samples, catheter tips, body fluids, and other clinical specimens). The published prevalence in earlier studies varied according to the studied population and the duration of research. In outpatient settings, the prevalence is reported to be between 0.11% and 0.75% [14]. In samples from inpatients, the prevalence was significantly higher, ranging from 0.39% to as much as 16.5% [14,15]. Variations in reports may be influenced by factors such as geographic location, the underlying clinical status of the tested individuals, and laboratory reporting protocols. The rise in the overall incidence of *Candida*, including *C. albicans* and NAC species, between 2016 and 2021 coincides with the introduction of MALDI-TOF in our laboratory in 2016. The enhanced accuracy of MALDI-TOF enabled the detection of isolates that may have been missed with earlier methods, leading to an apparent increase in reported positivity rates. However, the noticeable decline in candiduria cases in 2020 coincided with the COVID-19 pandemic and the associated closure of outpatient clinics, along with reduced inpatient services.

Consistent with the literature, *C. albicans* was the most frequently isolated species [14,16,17,18]. This observation is reassuring given its intrinsic susceptibility to all antifungal agents, as previously documented in our institution [13]. However, in 2013 and 2015, there was a shift in favor of NAC species. In 2013, yeast identification from urine cultures relied on conventional microscopic examination of budding and pseudohyphae plus germ tube testing. During this time, all *Candida*, except for *C. albicans*, were reported as “*Candida* species”. This raises the possibility that some isolates identified as NAC in 2013 might have been misidentified *C. albicans*.

*Candida tropicalis* was consistently the most frequently isolated NAC species in this study. It has been reported as the most prevalent *Candida* species in some regions [19,20]. Our results are consistent with data from North India, Cameron, Turkey, and Brazil but differ from trends observed in North America and Europe [19,20,21,22,23,24]. It was documented that *C. tropicalis* showed increased resistance to Flucytosine with emerging resistant to other antifungal classes, and therefore, continuous monitoring of these isolates is important [25].

The regression results from this study are consistent with global evidence indicating that female sex is a strong and consistent predictor of *Candida albicans* urinary tract infection. The association is commonly ascribed to anatomical predisposition, hormonal factors, and higher rates of antibiotic exposure in women [1]. It is believed that the prostatic fluid in males may exhibits antifungal activity against *Candida species* [26]. In females, elevated estrogen levels were linked to enhanced adherence of *Candida* to the genitourinary tract [27], making it a common colonizer in females. Moreover, frequent exposure to over-the-counter antifungal agents may have contributed to the prominent isolation of NAC species in samples obtained from our female population. The nearly two-fold increased odds observed in the present analysis mirror results from tertiary-care cohorts in Tanzania, where female predominance has been repeatedly documented [28].

In this cohort, samples from adults showed the highest amount of *Candida* during the period of the study. However, the association of candiduria with advanced age was the prevailing pattern over the years, aligning with previously published data [14,18,19]. It is likely that the number of *Candida* isolates in 2019 caused this rise in the overall number. The fluctuation observed in the dominant age group is probably related to the underlying risk factors of the studied population. Elderly patients are generally more vulnerable to infections due to their critical status and immunosuppression.

Most of our samples were obtained from hospitalized patients, with most being from those admitted to medical wards followed by intensive care units. Many are likely to be from indwelling urinary catheters, as this type of sample was observed to have the highest amount of *Candida*. It is well known that invasive procedures such as urinary catheters and intravenous central or peripheral venous lines facilitate *Candida* pathogenicity [29]. Biofilm production, for example, was more frequently associated with NAC species in patients with an indwelling urinary catheter [30]. It is important to highlight that in this study, *C. glabrata* and *C. krusei* were detected more frequently in samples from hospitalized patients. They were rarely seen in urine samples from patients attending clinics or emergency departments. These organisms are known for their resistance to fluconazole and reduced susceptibility to amphotericin B and other antifungal agents [8,9]. Prior exposure to antifungal agents during hospitalization may have influenced the frequency of these isolates. The strong influence of specimen type, especially the lower odds from random urine samples, supports the need for standardized collection methods for urine specimens. In this study, no emerging species were detected such as *Candida auris*. Other risk factors not considered in this work include length of stay in hospital, urogenital abnormalities, and diabetes mellitus.

The main limitation of this study is the absence of data on antifungal susceptibility testing. As per the routine laboratory practice at our institution, the reporting of yeasts from urine samples is limited to species identification. However, appreciation of the burden of NAC species in our population is worth highlighting to ensure the inclusion of reports from Saudi Arabia, where such data are currently lacking in the literature. Also, due to the retrospective nature of this study, clinical data retrieved from electronic systems were incomplete. Therefore, the current work was restricted to laboratory variables consistently available throughout the entire study period.

## 5. Conclusions

The present study highlighted the local prevalence and distribution of candiduria in a tertiary hospital over a decade of surveillance and associated risk factors. In clinical practice, considering species-level identification of *Candida*, accompanied by antifungal susceptibility testing, becomes crucial to allow for individualized therapeutic approaches, especially in complicated UTI cases.

## Figures and Tables

**Table 1:** Demographics and clinical characteristics of patients with candiduria.

Characteristics	Percentage (Frequency) 100.0% (n = 1667)	*p*-Value
**Age**		<0.05 *
Mean (SD)	53 (25.0)	
18 and less	6.7% (n = 111)	
19–60	48.7% (n = 811)	
61 and above	44.7 (n = 745)	
**Gender**		<0.05
Male to female ratio	1:2.5	
Female	71.4% (n = 1190)	
Male	28.6% (n = 477)	
**Location in the hospital**		<0.05
**Inpatient**	68% (n = 1141)	
Medical	35.5% (n = 405)	
Surgical	19.2% (n = 219)	
ICU	26.6% (n = 304)	
Pediatrics	4.9% (n = 56)	
Obstetrics and Gynecology	13.8% (n = 157)	
**Outpatients**	31% (n = 526)	
Clinics	32.3% (n = 170)	
Emergency	67.7% (n = 356)	
**Type of specimen**		<0.05
Urine—Bladder	5.5% (n = 91)	
Urine—Catheter	40.4% (n = 674)	
Urine—MSU	29.5% (n = 491)	
Urine—Random	24.7% (n = 411)	

Abbreviations: SD, standard deviation; D, day; Y, tear; ICU, intensive care unit; MSU, midstream urine. Statistical analysis was performed using the chi-square test. * *P* values below 0.05 were considered statistically significant.

**Table 2:** Univariate and multivariate regression analysis for the predictor of *Candida albicans*.

Factor	OR (95% CI)	*p*-Value	AOR (95% CI)	*p*-Value
**Age group**				
≤18 years	Ref		Ref	
19–60 years	0.75 (0.51–1.13)	0.165	0.75 (0.50–1.12)	0.165
>60 years	0.57 (0.46–0.69)	<0.001 **	0.56 (0.46–0.69)	<0.001 **
**Gender**				
Male	Ref		Ref	
Female	1.81 (1.46–2.25)	<0.001 **	1.81 (1.46–2.25)	<0.001 **
**Nationality**				
Saudi	Ref		Ref	
Non-Saudi	0.96 (0.76–1.23)	0.760	0.96 (0.76–1.23)	0.760
**Location**				
Inpatients	Ref		Ref	
Outpatients	1.09 (0.89–1.35)	0.375	1.09 (0.89–1.35)	0.375
**Specimen**				
Bladder	Ref		Ref	
Catheter	1.13 (0.72–1.78)	0.592	1.13 (0.72–1.79)	0.589
Midstream	1.25 (0.97–1.59)	0.080	1.26 (0.98–1.63)	0.071
Random	0.67 (0.51–0.88)	0.003 **	0.67 (0.51–0.88)	0.003 **

Adjusted for nationality and location. ** Significant at *p* < 0.05 level.

**Table 3:** Distribution of growth associated with candiduria across years, gender, type of specimen and hospital status.

Variable.	Description	Candiduria	*Candida albicans*, n (%)	*Non-albicans Candida*, n (%)
Year	2013	113 (7%)	36 (32%)	77 (68%)
	2014	131 (8%)	59 (46%)	72 (54%)
	2015	129 (8%)	56 (43%)	73 (57%)
	2016	132 (8%)	83 (63%)	49 (37%)
	2017	194 (12%)	125 (64%)	69 (36%)
	2018	169 (10%)	102 (60%)	67 (40%)
	2019	221 (13%)	141 (64%)	80 (36%)
	2020	179 (11%)	96 (54%)	83 (46%)
	2021	207 (12%)	115 (65%)	92 (35%)
	2022	129 (8%)	69 (53%)	60 (47%)
	2023	64 (4%)	38 (59%)	26 (41%)
Gender	Male	477 (29%)	213 (45%)	264 (55%)
	Female	1190 (71%)	707 (59%)	483 (41%)
Hospitalization	Inpatients	1144 (69%)	623 (54%)	521 (46%)
	Outpatients	523 (31%)	297 (57%)	226 (43%)
Specimen Type	Random	411 (25%)	225 (55%)	186 (45%)
	Midstream	491 (29%)	315 (64%)	176 (36%)
	Catheter	674 (40%)	332 (49%)	342 (51%)
	Bladder	91 (5%)	47 (52%)	44 (48%)
**Overall**		**1667**	**920**	**747**

## Data Availability

Data are available from the corresponding author on reasonable request, in accordance with privacy and ethical requirements.

## Supplementary Materials

The following supporting information can be downloaded at: https://www.mdpi.com/article/10.3390/medicina62050921/s1, Figure S1: Distribution of *Candida* species isolated from urine specimens in relation to patients’ (A) gender, (B) age, (C) hospitalization, and (D) type of urine specimen.

## Abbreviations

The following abbreviations are used in this manuscript:

NAC	Non-albicans *Candida*
UTI	Urinary Tract Infection
CLED	Cysteine–Lactose––Electrolyte-Deficient
CAN	Columbia Nalidixic Acid
MALDI-TOF	Matrix-Assisted Laser Desorption/Ionization Time-of-Flight
