# Supplementary material for: Species Distribution and Determinants of Candida Urinary Tract Infections: A 10-Year Retrospective Study in a Tertiary Hospital
Source: Medicina (Kaunas). 2026 May 9;62(5):921. doi: 10.3390/medicina62050921 (PMC13208417; doi:10.3390/medicina62050921)
Supplement: Supplementary file 1 [file medicina-62-00921-s001.zip › medicina-4283811-supplementary.pdf]

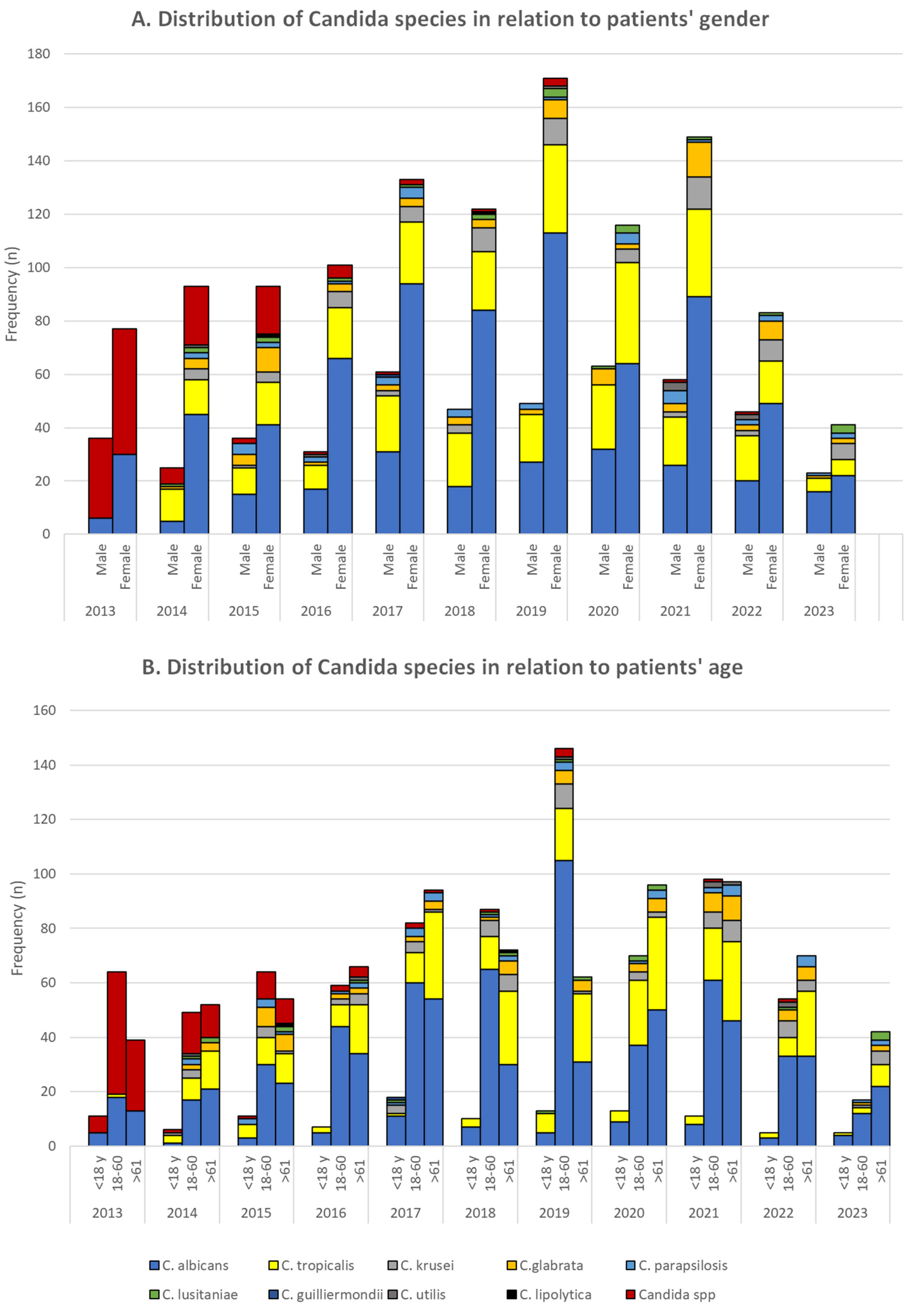
A. Distribution of Candida species in relation to patients' gender
Frequency (n)
0
20
40
60
80
100
120
140
160
180
Male
Female
2013
2014
2015
2016
2017
2018
2019
2020
2021
2022
2023
B. Distribution of Candida species in relation to patients' age
Frequency (n)
0
20
40
60
80
100
120
140
160
<18 y
18-60
>61
C. albicans
C. tropicalis
C. krusei
C.glabrata
C. parapsilosis
C. lusitaniae
C. guilliermondii
C. utilis
C. lipolytica
Candida spp

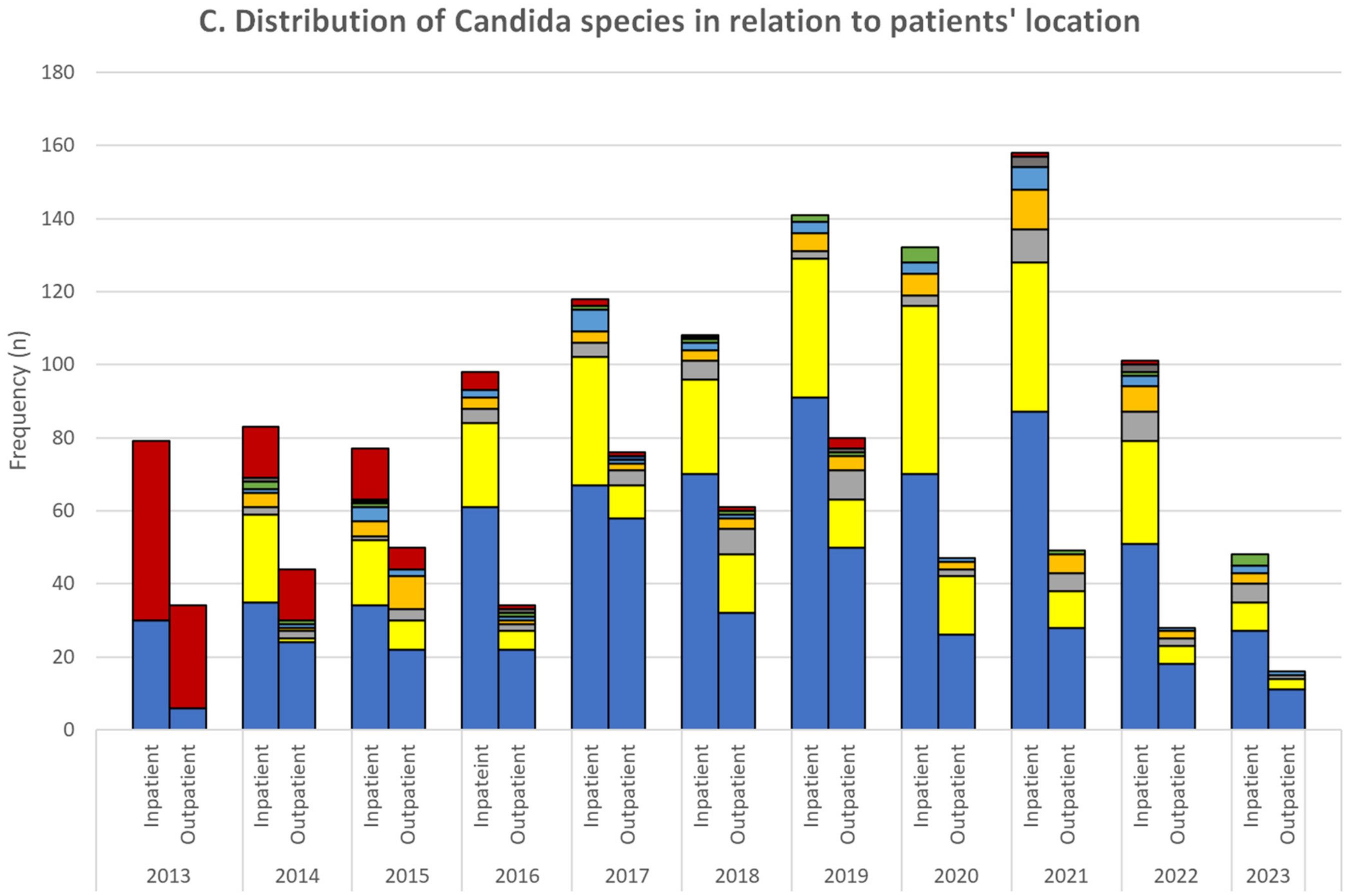
C. Distribution of Candida species in relation to patients' location
Frequency (n)
0
20
40
60
80
100
120
140
160
180
Inpatient
Outpatient
Inpateint
2013
2014
2015
2016
2017
2018
2019
2020
2021
2022
2023

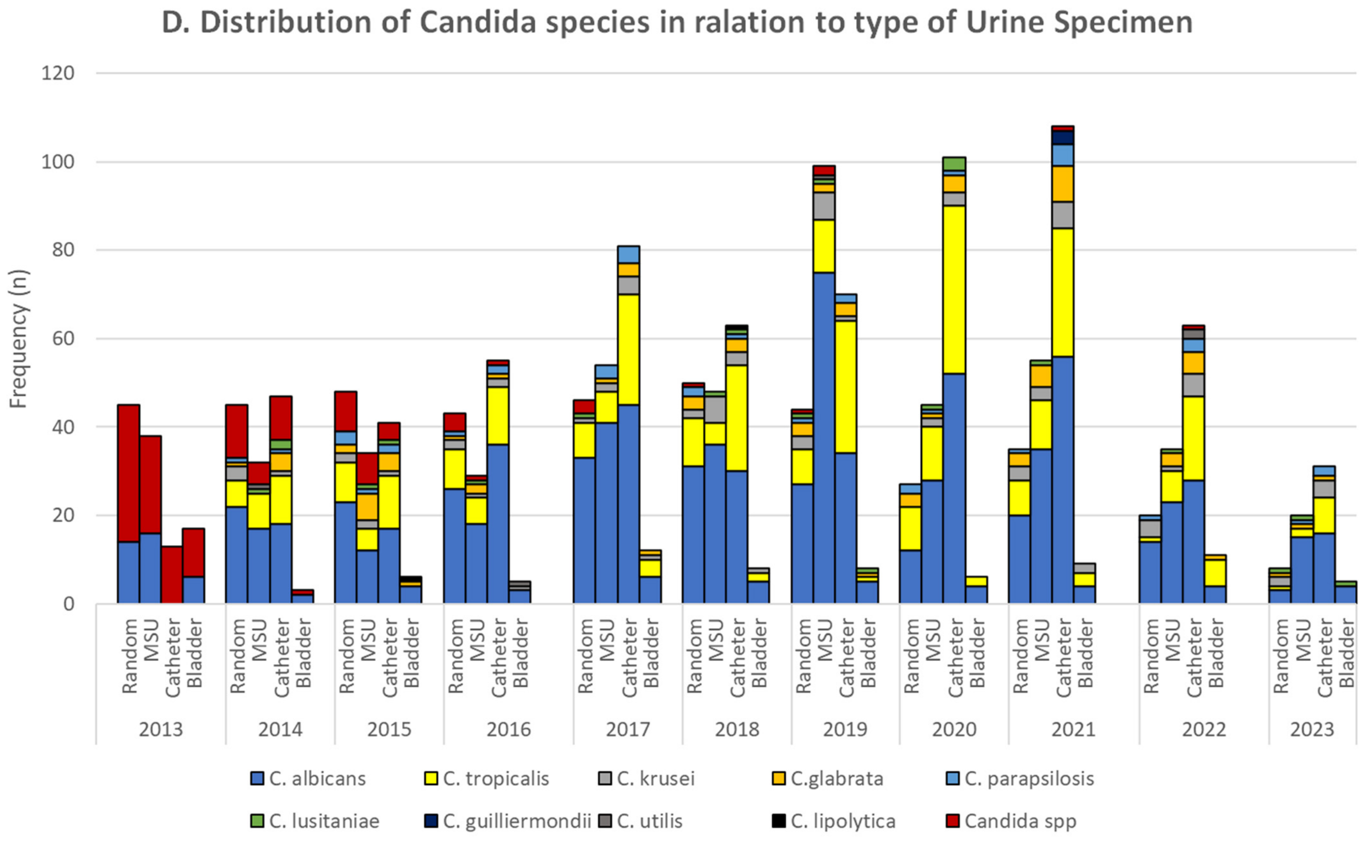
D. Distribution of Candida species in ralation to type of Urine Specimen
Frequency (n)
0
20
40
60
80
100
120
Random
MSU
Catheter
Bladder
2013
2014
2015
2016
2017
2018
2019
2020
2021
2022
2023
C. albicans
C. tropicalis
C. krusei
C.glabrata
C. parapsilosis
C. lusitaniae
C. guilliermondii
C. utilis
C. lipolytica
Candida spp

Figure 1S: Distribution of *Candida species* isolated from urine specimens in relation to patients' A) gender, B) age, C) hospitalization, and D) type of urine specimen.
